# Overcoming Therapeutic Resistance of Triple Positive Breast Cancer with CDK4/6 Inhibition

**DOI:** 10.1155/2018/7835095

**Published:** 2018-06-19

**Authors:** Troy B. Schedin, Virginia F. Borges, Elena Shagisultanova

**Affiliations:** Young Women's Breast Cancer Translational Program, University of Colorado Denver, 13001 E 17th Pl, Aurora, CO 80045, USA

## Abstract

Triple positive breast cancers overexpress both the human epidermal growth factor receptor 2 (HER2) oncogene and the hormonal receptors (HR) to estrogen and progesterone. These cancers represent a unique therapeutic challenge because of a bidirectional cross-talk between the estrogen receptor alpha (ER*α*) and HER2 pathways leading to tumor progression and resistance to targeted therapy. Attempts to combine standard of care HER2-targeted drugs with antihormonal agents for the treatment of HR+/HER2+ breast cancer yielded encouraging results in preclinical experiments but did improve overall survival in clinical trial. In this review, we dissect multiple mechanisms of therapeutic resistance typical of HR+/HER2+ breast cancer, summarize prior clinical trials of targeted agents, and describe novel rational drug combinations that include antihormonal agents, HER2-targeted drugs, and CDK4/6 inhibitors for treatment of the HR+/HER2+ breast cancer subtype.

## 1. Triple Positive Breast Cancer Displays Increased Resistance to Targeted Therapy

Breast cancer is the most frequently diagnosed cancer, excluding skin malignancies, and a second leading cause of cancer death in women in the United States [1]. Approximately 20% of breast cancers overexpress the human epidermal growth factor receptor 2 (HER2), a transmembrane tyrosine kinase receptor mediating cell growth, differentiation, and survival [2]. HER2-positive (HER2+) breast tumors are more aggressive and historically have been associated with poorer outcomes compared to HER2-negative (HER2-) tumors, although the introduction of HER2-targeted therapies has allowed for significant improvements in survival of patients with HER2+ breast cancer [3–5].

Approximately half of HER2+ breast tumors overexpress hormone receptors (HR) [6, 7]. These cancers represent a therapeutic challenge because of a bidirectional cross-talk between HR and HER2 pathways leading to tumor progression and resistance to targeted therapies [8, 9]. HR+/HER2+ tumors are sometimes called “triple positive,” if HER2 and both estrogen receptor (ER) and progesterone receptor (PR) are expressed. Less frequently, HR+/HER2+ tumors express only one of the hormonal receptors (either ER or PR). In the University of Colorado Denver clinical database, among 114 HR+/HER2+ cases, 71% were triple positive, 21% were ER+/PR-/HER2+, and 8% were ER-/PR+/HER2+. Differences in clinical behavior between HER2 amplified tumors that are ER+/PR+, ER+/PR-, or ER-/PR+ are not well studied. It is assumed that even if only one of the hormonal receptors is expressed, tumor proliferation is driven by HR signaling along with HER2 pathway, which may lead to a resistant phenotype. Another complicating factor is that HR+/HER2+ breast tumors are heterogeneous at a molecular level and HR status does not completely recapitulate this heterogeneity: 40-50% of these tumors belong to HER2-enriched PAM50 molecular subtype, while the rest are classified as luminal A or B subtype [10, 11]. Intrinsic molecular subtypes of HR+/HER2+ breast cancer may affect therapeutic sensitivity [11, 12], as discussed below.

Multiple studies have shown that HR expression confers resistance to HER2-targeted therapies [8, 13, 14]. In HR+/HER2+ breast cancer cell lines, ER signaling and ER transcriptional activity are upregulated following treatment with HER2-targeted agents trastuzumab and lapatinib and ER functions as the key survival pathway reducing sensitivity to HER2-blockade [13, 15, 16]. The same phenomenon is seen in patients on neoadjuvant treatment. Multiple prospective clinical trials demonstrated that pCR rates in patients with HR+/HER2+ breast tumors are 1.5 – 2.5 times lower compared to patients with HR-/HER2+ tumors, regardless of HER2-targeted and chemotherapeutic agents administered (Table 1). Preclinical and clinical studies in HER2+ metastatic breast cancer confirmed that expression of HR is associated with reduced responsiveness to trastuzumab [13, 14], and combinations of antihormonal and HER2-targeted agents led to progression free survival (PFS) benefits in some trials [17–19].

Similarly, HER2 overexpression is a major determinant of resistance to endocrine therapy [7, 20–22]. HR+ breast cancer cell lines that are sensitive to tamoxifen acquire tamoxifen resistance after transfection with the HER2 oncogene [20, 22]. Analysis of tumor samples from postmenopausal patients with stages II and III HR+ breast cancer treated on two-independent neoadjuvant endocrine therapy trials showed that HR+/HER2+ tumors had significantly higher histologic grade and Ki-67 and significantly less suppression of Ki-67 after treatment with tamoxifen or an aromatase inhibitor (AI) compared with HR+/HER2- tumors. These tumors display continued estrogen-independent proliferation despite ongoing endocrine therapy [23]. Results of two-adjuvant therapy clinical trials (Breast International Group 1-98 study and the Arimidex or Tamoxifen Alone or in combination study) demonstrated that HER2+ status is associated with a significantly higher relapse rate, regardless of whether the adjuvant antihormonal therapy administered was tamoxifen or an AI [24, 25]. Similarly, studies in metastatic breast cancer demonstrated decreased responses to antihormonal therapy in patients with HR+/HER2+ tumors [23, 25–28].

NCCN guidelines (version 4.2017) suggest several options for an initial treatment of HR+/HER2+ metastatic disease. Chemotherapy with a taxane plus trastuzumab and pertuzumab remains a preferred frontline regimen based on the CLEOPATRA clinical trial [5]. NCCN included the antibody-drug conjugate TDM-1 as one of the frontline options after considering the results of the MARIANNE trail [29]. Other options include single agent endocrine therapy (for patients with bone or soft tissue metastases, or asymptomatic minimal visceral disease), or dual combinations of antihormonal and HER2-targeted agents [30]. Although the preferred combination chemotherapy approach is highly effective, it is associated with multiple side effects. Single agent antihormonal therapy generally has a poor efficacy in patients with HR+/HER2+ breast cancer, resulting in PFS of 3-4 months [19, 31]. Dual combinations of HER2-targeted and antihormonal agents demonstrated efficacy in phase II clinical trials [17, 18]; however, they did not improve overall survival in randomized phase III clinical trials [19, 31]. Therefore, there is an unmet clinical need to develop a more effective chemotherapy-free approaches based on novel targeted drug combinations for patients with HR+/HER2+ breast cancer.

Below we summarize current approaches to targeted therapy in HR+/HER2+ breast cancer, highlight drug resistance mechanisms, and focus on CDK4/6 inhibitors as promising agents that may counteract therapeutic resistance in patients with HR+/HER2+ breast cancer.

## 2. Dual Blockade: Combining Antihormonal and HER2-Targeted Agents

Preclinical modeling in breast tumor cell lines and murine xenografts demonstrated synergy of HER2-targeted agents combined with endocrine therapy in suppressing growth of HR+/HER2+ breast tumors [13, 59]. However, translation of these exciting preclinical results into human clinical trials has not been straightforward.

Neoadjuvant randomized phase III clinical trial NSABP B-52 explored the concept of dual targeting of HER2 and HR pathways combined with chemotherapy, with the goal of improving pCR rates in patients with HR+/HER2+ early breast cancer. In this trial, 308 women were randomized to receive neoadjuvant chemotherapy with docetaxel, carboplatin, trastuzumab, and pertuzumab (*n* = 154), or the same chemotherapy plus endocrine therapy with estrogen deprivation (*n* = 157). The pCR rates were numerically better in the estrogen deprivation arm comparing to control (46% versus 41%); however, the difference did not reach statistical significance (*p* = 0.39). A subgroup analysis looking at patients by menopausal status showed no significant difference for premenopausal (46% versus 44%) or postmenopausal women (45% versus 38%) [75].

Phase II neoadjuvant trial PAMELA enrolled 151 patients with stage I-IIIA HER2+ breast cancer [10]. The trial was specifically designed to test the hypothesis that PAM50 tumor molecular subtypes will determine response to targeted therapy. All patients received lapatinib and trastuzumab for 18 weeks. Additionally, patients with HR+/HER2+ disease received daily letrozole or tamoxifen. The overall pCR rate in the breast was 30.2% (40.2% in HER2-enriched tumors irrespective of HR status versus 10.0% in non-HER2-enriched tumors). HR status lost its association with pCR once intrinsic molecular subtypes were taken into account in the multivariable model. Therefore, this trial suggested that the HER2-enriched subtype is a predictor of anti-HER2 sensitivity, regardless of HR status [10, 11]. One striking peculiarity of the trial results was the low pCR rate in patients with luminal tumors despite dual HR and HER2 blockade.

In metastatic settings, the eLEcTRA trial compared efficacy of letrozole combined with trastuzumab (*N* = 26) versus letrozole alone (*N* = 31) as a frontline treatment [17]. Median time to progression was 3.3 months in the letrozole group compared to 14.1 months in the trastuzumab and letrozole group. Clinical benefit rate was 39% compared to 65% in the single agent letrozole versus dual combination. The trial showed that the combination of letrozole and trastuzumab may be a safe and effective treatment option. However, although this was a randomized trial, the sample size was quite small.

Results of two larger randomized phase III clinical trials combining antihormonal therapy with HER2-targeted agents for metastatic breast cancer have been reported [19, 31]. The TAnDEM trial evaluated the benefit of adding trastuzumab to anastrozole as a frontline therapy in 207 patients with HR+/HER2+ metastatic breast cancer. Median PFS was 4.8 months for the combination group versus 2.4 months for the anastrozole monotherapy group, with a hazard ratio of 0.63 (*p* = 0016; 95% CI, 0.47 to 0.84). In patients with centrally confirmed HR+ tumors, median PFS was 5.6 versus 3.8 month in the trastuzumab plus anastrozole and anastrozole alone arms, respectively (*p* = 0.006). The overall response rate (ORR) was significantly higher with the combination treatment compared with anastrozole alone (20.3%* v* 6.8%; *p* = 018). The clinical benefit rate (CBR) was also higher in patients in the combination arm compared with the anastrozole arm (42.7%* v* 27.9%; *p* = 0.026). No statistically significant improvement in overall survival (OS) was demonstrated (28.5* v* 23.9 months for dual combination versus monoagent letrozole; *p* = 0.325) [31].

Similarly, in the EGF30008 study, anti-HER2 tyrosine kinase inhibitor lapatinib was combined with letrozole and compared to letrozole plus placebo in 219 patients with HR+ metastatic breast cancer. In the HER2+ subgroup, the addition of lapatinib reduced risk of disease progression, with a hazard ratio of 0.71 (*P* = 0.019; 95% CI, 0.53 to 0.96) and median PFS of 8.2 versus 3.0 months. The ORR was also higher in the combination therapy group (28%* v* 15%; *P* = 0.021). CBR was significantly greater for lapatinib plus letrozole (48%* v* 29%; odds ratio 0.4; 95%CI, 0.2 to 0.8; *P* = 0.03). These benefits did not translate into an improvement in median OS (33.3* v* 32.3 months) [19].

The effect of combined HR and HER2 blockade was further evaluated in the PERTAIN randomized phase II clinical trial. In this trial, 258 postmenopausal patients with metastatic HR+/HER2+ breast cancer who did not receive prior systemic chemotherapy for metastatic disease were randomized to receive a combination of trastuzumab and an AI (anastrozole or letrozole), or trastuzumab plus pertuzumab and an AI. Fifty-seven percent of patients initially received 18-24 weeks of induction chemotherapy with docetaxel or paclitaxel in combination with HER2-targeted agents. The addition of pertuzumab led to a statistically significant increase of median PFS from 15.8 months to 18.9 months (trastuzumab + AI versus trastuzumab + pertuzumab + AI, HR 0.65, 95%CI 0.48–0.89; *p* = 0.007) [18]. These results are drastically different from the TAnDEM trial, where patients on trastuzumab and an AI had a median PFS of 4.8 months [31]. One potential explanation could be that the TAnDEM trial enrolled “all comers” for a frontline targeted therapy, while in the PERTAIN trial more than half of the patients, potentially those with more aggressive disease, received induction chemotherapy prior to going on targeted therapy maintenance. Patients who did not receive induction chemotherapy had much better outcomes with the HER2 and HR blockade compared to the TAnDEM trial. However, it is not clear if these patients actually had less aggressive disease, because the decision of whether or not to administer induction chemotherapy was at the discretion of the treating physician; therefore, selection bias might have been introduced. Pertuzumab, trastuzumab and an AI combination was well tolerated, making this an attractive treatment option for a selected patient population. The trial clearly demonstrated that pertuzumab and trastuzumab maintenance is better than trastuzumab alone. The PERTAIN study did not address the question of whether the addition of endocrine therapy to dual HER2-blockade further improves efficacy, because both randomization groups received an AI.

Although the TAnDEM and EGF30008 studies showed statistically significant improvement of PFS in patients with HR+/HER2+ tumors with the addition of HER2-targeted agents to endocrine therapy, these phase III trials were not practice changing because PFS benefits were small and no benefits in OS were demonstrated. What is not known is whether these results were due to patient selection, limitations of specific HER2-targeted agents, inherent resistance mechanisms that were not counteracted by antihormonal and HER2-targeted therapy, or a combination of all these factors. The PERTAIN trial showed feasibility of a frontline multiagent targeted therapy approach in selected patients, although these results may not be fully applicable to the overall population of patients with HR+/HER2+ disease. New rationally designed combinations of targeted agents for patients with HR+/HER2+ breast cancer are warranted.

## 3. CDK4/6 Inhibitors Synergize with Antihormonal and HER2-Targeted Agents

Inhibition of the cyclin D1-CDK4/6 complex emerged as a promising therapeutic strategy in breast cancer. In a pivotal study of the CDK4/6 inhibitor palbociclib, Finn and colleagues [37] compared baseline gene expression profiles from breast cancer cell lines highly sensitive or resistant to palbociclib. HR+ cell lines, including those with* HER2* amplification, were the most sensitive, and there was a significant overlap between the gene expression profiles associated with palbociclib sensitivity and that which distinguished a luminal breast cancer subtype [37]. In preclinical studies, palbociclib was active against both luminal A and luminal B tumors and synergized with both tamoxifen and anti-HER2 agents (trastuzumab, lapatinib, and TDM-1) providing a potent addition to antihormonal and HER2-targeted therapies [37, 76, 77]. Additionally, another CDK4/6 inhibitor, abemaciclib, showed significant activity in HER2+ preclinical models, supporting the hypothesis that CDK4/6 inhibitors may resensitize resistant tumors to the HER2 blockade [78].

Palbociclib was FDA approved in patients with HR+ metastatic breast cancer based on the results of the PALOMA-2 randomized phase II clinical trial, which showed marked improvement in median PFS in women who received palbociclib and letrozole versus letrozole alone (26.1 versus 7.5 months) [38]. Additionally, palbociclib demonstrated remarkable activity in the second-line metastatic setting in combination with fulvestrant in the PALOMA-3 clinical trial, resulting in more than a doubling of median PFS (9.2 months palbociclib with fulvestrant versus 3.8 months placebo with fulvestrant; HR 0.42; *P* <0.001) [39]. Comparable efficacy in patients with HR+/HER2-metastatic breast cancer was demonstrated for ribociclib and letrozole combination in the MONALEESA-2 trial [79] and for abemaciclib and antihormonal therapy combination in the MONARCH-2 and MONARCH-3 clinical trials [80, 81]. Notably, abemaciclib has a remarkable single agent activity [82] and documented efficacy in the central nervous system metastatic disease [83].

Taken together, this data suggest that it is reasonable to combine HER2-targeted agents with synergistic combinations of CDK4/6 inhibitors and antihormonal agents for the treatment of patients with HR+/HER2+ breast cancer. Multiple clinical trials have shown synergy of CDK4/6 inhibitors with endocrine therapy, and large amounts of preclinical data support synergy of CDK4/6 inhibitors with HER2-targeted therapies. Triple targeting of HR, HER2, and CDK4/6 pathways is a promising approach that has a strong preclinical rationale. This approach is now being tested in clinical trials.

## 4. Triple Blockade of HR, HER2, and Cell Cycle Checkpoints: Signaling Rationale and Ongoing Clinical Trials

**Figure 1:**
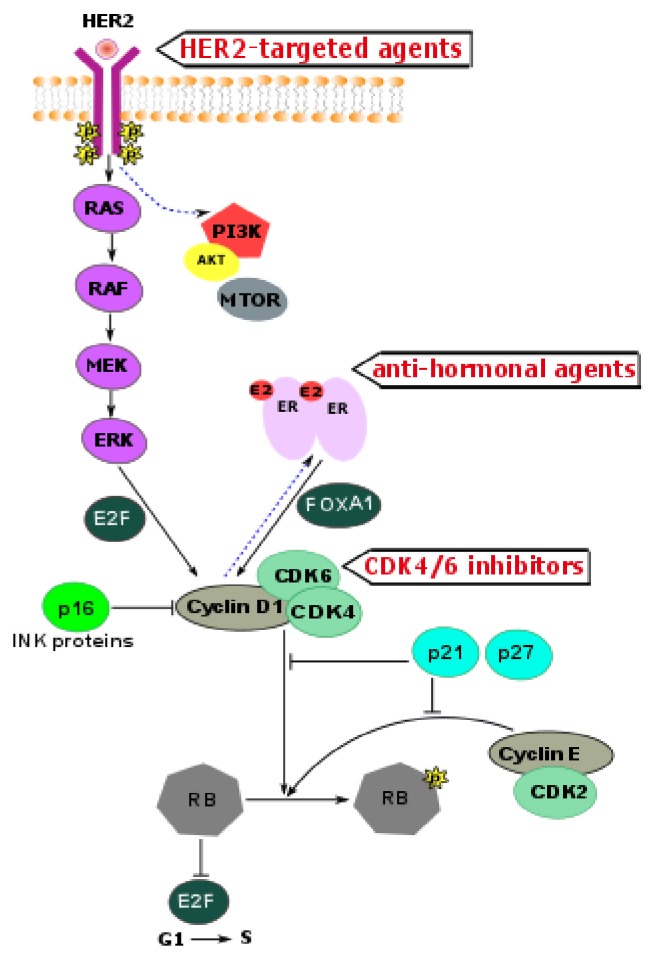
HR and HER2 signaling converge at cell cycle checkpoints.

Activation of cyclin D1 and CDK4/6 plays a significant role in the tumorigenesis of HR+/HER2+ breast cancer [37, 76, 84]. Mitogenic signaling from HER2 and HR receptors converges at cell cycle checkpoints and results in the synergistic increase of cyclin D1 expression (Figure 1). Specifically, HER2/MAPK kinase signaling activates E2F transcription factors, leading to the transcription of* CCND1* gene encoding cyclin D1, while active estrogen receptor alpha (ER*α*) in complex with FOXA1 transcription factor intensifies* CCND1* transcription through an estradiol-responsive enhancer [85].* CCND1* gene located on chromosome 11q13 is amplified in ~15% of breast cancers [86, 87]. However, cyclin D1 is overexpressed at the protein levels in ~50% of breast cancers in the presence or absence of gene amplification [88]. The difference in the frequency of* CCND1* gene amplification and protein overexpression can be explained at least in part by the* CCND1* promoter activation by aberrant mitogenic signaling in tumors with HER2 amplification or overexpression [89]. Consistent with this data, the frequency of cyclin D1 overexpression is two times higher in luminal B versus luminal A tumors (58% versus 29%) [90], because many of luminal B tumors are HER2 amplified. Amplification or overexpression of cyclin D1 is strongly associated with short survival in breast cancer patients [91].

Cyclin D1–CDK4/6 complex phosphorylates the retinoblastoma protein (RB). Controlled phosphorylation and deactivation of RB is essential for progression of the cell cycle from G1 to S phase [88]. Activity of the cyclin D1–CDK4/6 is counteracted by tumor suppressor protein p16 and other INK-family proteins. However, p16 is frequently inactivated in breast tumors [85]. Notably, cyclin D1 associates with ER*α* and the steroid receptor coactivator increasing transcriptional activity of ER*α* [92–94].

In addition to being catalytically active, the cyclin D1–CDK4/6 complex sequesters the cell cycle inhibitors p21 and p27, thereby promoting the activation of another key component of G1 to S transition: the cyclin E–CDK2 complex. This complex can further phosphorylate RB, leading to full saturation of all phosphorylation sites. Hyperphosphorylated RB loses its inhibitory effect on the E2F transcription program, allowing G1 to S transition [95, 96]. Because cyclin E itself is an E2F target gene, cyclin E may reinforce its own expression. Once cyclin E–CDK2 becomes active, RB phosphorylation is rendered partially independent of the mitogenic control that regulates cyclin D1 expression [97]. Additionally, CDK2 phosphorylates ER*α*, providing a positive feedback loop and further increasing ER*α* transcriptional activity [98].

Considering the synergistic effects of ER*α* signaling and HER2 overexpression on cell cycle checkpoints, as well as multiple feedback loops between these pathways, there is a strong signaling rationale to combine CDK4/6 inhibitors with HER2 inhibitors and antihormonal agents for treatment of HR+/HER2+ breast cancer. Multiple clinical trials are ongoing in Europe and the United States to test triple combinations of HR, HER2, and CDK4/6 inhibitors (Table 2).

The goal of these clinical trials is to develop an effective and safe targeted therapy-based regimen which will overcome multiple drug resistance mechanisms typical of HR+/HER2+ breast cancer, resulting in the improved response to neoadjuvant treatment in early disease, as well as prolonged PFS and improved quality of life in metastatic settings. Potentially overlapping side effects of the drugs in combination could be of concern, especially diarrhea, which is a side effect of CDK4/6 inhibitors and many HER2-targeted agents. However, overall safety and toxicity profiles of CDK4/6 inhibitors, HER2-targeted drugs, and antihormonal agents are favorable, and the majority of side effects are nonoverlapping. Targeted drug combinations are attractive from patient perspective, because they are expected to be tolerated significantly better compared to chemotherapy-based combinations.

## 5. Potential Mechanisms of Resistance to HR and HER2 and Cell Cycle Checkpoint Inhibition

In the design of rational drug combinations, it is critical to evaluate pathways of resistance to each targeted agent and identify potential cross-resistance mechanisms. Mathematical modeling of tumor clonal evolution demonstrated that even a single genetic alteration conferring resistance to two-targeted agents may decrease efficacy of treatment with these agents given in combination. Similarly, once metastases have been established with significant tumor burden, the probability of mutation(s) that will confer multidrug resistance increases and treatment is doomed to fail [99]. Will the combinations of HER2-targeted agents with antihormonal drugs and CDK4/6 inhibitors avoid this problem? To answer this question, we performed a literature search looking for the mechanisms of resistance to antihormonal agents, HER2 inhibitors, and CDK4/6 inhibitors in published preclinical and clinical studies. Our goal was to identify candidate single mechanism, which could confer resistance to all three drugs in combination. This exploratory analysis was not intended to provide guidance in clinical management, but rather to provide a framework for further studies. The results of our findings are summarized in Table 3.

As evident in Table 3, there are multiple potential mechanisms of resistance to single agents and significantly fewer pathways of resistance to dual and triple targeted drug combinations. Cyclin E1 amplification or overexpression potentially could confer resistance to all three drugs in our combination of interest (anti-endocrine agents, HER2-targeted drugs, and CDK4/6 inhibitors). Amplification of* CCNE1* gene encoding cyclin E1 is uncommon in HR+/HER2+ cancer subtype [100], although overexpression of cyclin E1 is a more common event [101]. Cyclin E1 amplification and overexpression have been shown to mediate resistance to antiendocrine and HER2-targeted agents in patients [44, 45]. Resistance to CDK4/6 inhibitors due to cyclin E1 overexpression has been demonstrated in preclinical models [46]; however, it was not yet confirmed in the clinic. Whether cyclin E1 amplification or overexpression could modulate patient response to CKD4/6 inhibitors remains to be determined. Assessment of cyclin E1 levels in clinical studies of HR, HER2, and CDK4/6 inhibitors is certainly of great interest.

CDK2 inhibitors might provide the ability to target tumors driven by cyclin E1 amplification [45, 102]. Several nonselective CDK inhibitors capable of targeting CDK2 were tested in clinical trials; however, clinical development was stopped because of toxicity [103]. Selective CDK2 inhibitors are not yet clinically available, although there is a great interest in development of this compounds.

Based on nonoverlapping resistance mechanisms, other rational drug combinations for treatment of HR+/HER2+ breast cancer may be suggested (for example, a combination of antihormonal drugs with HER2-targeted agents and PI3K or mTOR inhibitors). Some of these combinations are now being tested in clinical trials, which is outside of the scope of this paper.

In conclusion, triple combination targeting therapy (HER2 and CDK4/6 inhibitors combined with antihormonal therapy) remains promising in HR+/HER2+ breast cancer, given largely nonoverlapping resistance mechanisms to the agents in this combination. An overlapping mechanism of resistance to all three drugs probably exists (cyclin E overexpression). Assessment of this potential resistance mechanisms should be performed in the ongoing clinical trials.

## 6. Concluding Remarks

Breast tumors with HR expression and HER2 amplification represent a therapeutic challenge because they utilize multiple oncogenic drivers and pathways of therapeutic resistance. Based on the vast amount of preclinical and clinical data, the concept of triple targeting of HR, HER2, and CDK4/6 pathways simultaneously is a logical approach. Therapy with a triple combination of agents blocking HR, HER2, and CDK4/6 is supported by a strong signaling rationale and is feasible from a toxicity standpoint. With a majority of the resistance mechanisms being nonoverlapping, this promising combination has a reasonable potential to be effective and to overcome targeted therapy resistance. Multiple clinical trials are underway to test triple combinations of antihormonal therapy with HER2 and CDK4/6-targeted agents in locally advanced and metastatic HR+/HER2+ breast cancer, offering new hope to patients with this challenging disease.

## Figures and Tables

**Table 1:** Pathologic complete response after neoadjuvant chemotherapy in patients with HER2-positive breast cancer stratified by hormonal receptor status.

**Clinical trial**	**Chemotherapy and HER2-targeted agents**	**Outcome**	**pCR rate** **HR+/HER2+**	**pCR rate** **HR-/HER2+**	**Reference**
NeoSphere	Docetaxel Carboplatin Trastuzumab	ypT0^*a*^	20.0%	36.8%	[32]

NeoSphere	Docetaxel Carboplatin Trastuzumab Pertuzumab	ypT0	26.0%	63.2%	[32]

NeoALTO	Paclitaxel Lapatinib Trastuzumab	ypT0	41.6%	61.3%	[33]

NOAH	Doxorubicin Paclitaxel Cyclophosphamide Methotrexate Fluorouracil Trastuzumab	ypT0 ypN0^*b*^	30.0%	51.2%	[12]

ACOSOG Z1041	5-fluorouracil Epirubicin Cyclophosphamide Paclitaxel Trastuzumab	ypT0	47.6%	70.4%	[34]

*a*–ypT0: pathologic complete response in the breast (absence of invasive neoplastic cells);* b*–ypN0: pathologic complete response in the axillary lymph nodes (absence of invasive neoplastic cells).

**Table 2:** Ongoing clinical trials combining CDK4/6 inhibitors with HER2-targeted and antihormonal agents in patients with HR+/HER2+ breast cancer.

**Clinical trial**	**Therapeutic agents**	**Phase **	**Line of therapy** **Disease stage**	**Location**
NCT03054363 ^*a*^	(i) Palbociclib (ii) Tucatinib (iii) Letrozole	Ib/II	1^st^ line and beyond Stage IV	United States ABRCC consortium

NCT02947685 (PATINA)	(i) Palbociclib (ii) Trastuzumab (iii) Pertuzumab (iv) Any AI^*b*^ or fulvestrant	III	1^st^ line maintenance after 4-8 cycles of induction chemotherapy Stage IV	United States Alliance foundation

NCT02907918 (PALTAN)	(i) Palbociclib (ii) Trastuzumab (iii) Letrozole	II	Neo-adjuvant therapy Stage II, III	United States

NCT02675231 (MonarcHER2)	(i) Abemaciclib (ii) Trastuzumab (iii) Fulvestrant	II	3^rd^ line and beyond Stage IV	Worldwide

NCT02448420 (PATRICIA)	(i) Palbociclib (ii) Trastuzumab (iii) Letrozole	II	3^rd^ to 5^th^ line of therapy Stage IV	Spain SOLTI group

NCT02530424 (NA-PHER2)	(i) Palbociclib (ii) Trastuzumab (iii) Pertuzumab (iv) Fulvestrant	II	Neo-adjuvant therapy Stage I-III	Italy

NCT03304080	(i) Palbocilib (ii) Trastuzumab (iii) Pertuzumab (iv) Anastrozole	I/II	1^st^ line Stage IV	United States

*a*: clinical trial number in the US National Institutes of Health clinical trial database; *b*–AI: aromatase inhibitor.

**Table 3:** Potential pathways of resistance to antihormonal agents, HER2 inhibitors and CDK4/6 inhibitors.

**Mechanisms**	**Resistance to therapy**	**Counteracted by**
**Cell cycle checkpoints pathway**		

Cyclin D1 amplification or overexpression	Endocrine therapy (P, C)^*a*^ [35, 36]	CDK4/6 inhibitors [37–40]

CDK4 amplification	CDK4/6 inhibitors (P) [41, 42]	To be studied

CDK6 amplification	CDK4/6 inhibitors (P)^*b*^ [43]	To be studied

Cyclin E1 amplification or overexpression	Endocrine therapy (P, C) [44] HER2 inhibitors (P, C) [45] CDK4/6 inhibitors (P) [46]	CDK2 inhibitors [45]

Cyclin E2 amplification or overexpression	Endocrine therapy (P) [47]	CDK2 inhibitors [47]

RB loss	CDK4/6 inhibitors (P) [37, 48]	To be studied

p21 loss	Endocrine therapy (P, C) [49, 50] CDK4/6 inhibitors (P) [51]	To be studied

p27 loss	Endocrine therapy (P, C) [50, 52] CDK4/6 inhibitors (P) [51]	To be studied

$\underline{ER\alpha \;\;pathway}$		

ESR1 activating mutations	Endocrine therapy (P, C) [53–55]	CDK4/6 inhibitors [56] mTOR inhibitors [57] Fulvestrant [56] Novel ER antagonists [58]

**MAPK kinase pathway**		

HER2 amplification	Endocrine therapy (P, C) [7, 20–28]	HER2 inhibitors [13, 59]

HER2 truncation (p95HER2) or mutations in the extracellular domain	HER2-targeted antibodies (P, C) [60, 61]	HER2 small molecule inhibitors [61]

HER3 amplification	HER2 inhibitors [62]	HER3 inhibitors [63–65]

C-MYC overexpression	Endocrine therapy (P) [66]	CDK1 inhibitors [67, 68]

**PI3K/AKT/mTOR pathway**		

PI3K pathway activation	Endocrine therapy (P, C) [69] HER2 inhibitors (P) [62, 70, 71]	PI3K inhibitors [72] mTOR inhibitors [62, 71, 73] CDK4/6 inhibitors [74]

*a*–P: resistance demonstrated in preclinical studies; C: resistance shown in clinical studies; b: amplification of CDK6 has been linked to both sensitivity [41] and resistance [43] to CDK4/6 inhibitors; additional studies are needed.
